# Comparison of the Weil and Triple Weil Osteotomies: A Clinical Retrospective Study

**DOI:** 10.7759/cureus.22220

**Published:** 2022-02-14

**Authors:** Dimitrios Bougiouklis, Minos Tyllianakis, Despoina Deligianni, Elias Panagiotopoulos

**Affiliations:** 1 Orthopaedics and Traumatology, General Hospital of Ammochostos, Paralimni, CYP; 2 Department of Orthopaedics and Traumatology, University General Hospital of Patras, Patra, GRC; 3 Department of Mechanical Engineering and Aeronautics, University of Patras, Patra, GRC; 4 Department of Orthopaedics and Traumatology, University of Patras, Patra, GRC

**Keywords:** results, complications, triple weil osteotomy, weil osteotomy, propulsive metatarsalgia, third rocker metatarsalgia

## Abstract

Introduction: The Weil and triple Weil osteotomies are two widely used procedures in the surgical treatment of metatarsalgia. The aim of this comparative retrospective study was to evaluate the functional results and determine the complications of the two types of osteotomies in a series of patients who underwent surgery due to third rocker metatarsalgia.

Material and methods: In this paper, 71 patients were included between September 2015 and October 2020. The average age was 58 years old (age range: 28-72). Of all the patients, 27 suffered from metatarsalgia due to systemic (extra-regional) or regional diseases were excluded. The remaining 44 patients, after six months of unsuccessful conservative treatment, underwent surgery. Based on the preoperative planning to restore the peripheral parabolic curve of the metatarsals, when a shortening of less than or equal to 3 mm was required, a Weil osteotomy was performed. However, when a shortening of more than 3 mm was required, a triple Weil osteotomy was performed. Therefore, two groups of patients were formed, and a total of 90 osteotomies were performed. During the postoperative period, all the patients were clinically and radiographically assessed. The American Orthopedic Foot and Ankle Society (AOFAS) score was used for the assessment of the functional result, while the pain was assessed using the Visual Analogue Scale* *(VAS).

Results: The mean follow-up was 24 months. The average operative time for the Weil and the triple Weil osteotomies was 22.8 minutes and 31.5 minutes, respectively. In group A, preoperatively, the average AOFAS score was 31/100, and postoperatively, it was 89/100. In group B, the corresponding values were 30/100 and 93/100, respectively. In group A, the preoperative VAS score was 7.8/10, while the postoperative VAS score was 1.3/10. In group B, the corresponding values were 8.2/10 and 1.7/10, respectively. In group A, stiffness had a percentage equal to 60.9%, and a floating toe was noticed in 16 osteotomies. In group B, superficial infection represented the commonest complication, with an incidence of 25.6%.

Conclusion: Both Weil and triple Weil osteotomies are effective procedures in the surgical treatment of patients who suffer from third-rocker metatarsalgia. In both cases, correct preoperative planning is of paramount importance for the outcome. However, in terms of the appearance of the floating toe, it seems that in cases where a ray’s shortening of more than 3 mm is required, the triple osteotomy is superior to the Weil osteotomy.

## Introduction

In the second half of the last century, surgical treatment of metatarsalgia was associated with poor results [1]. Initially, the lack of a clear distinction between the different types of metatarsalgia led to the use of the same procedure in all cases. Consequently, in many cases, the unpredictable shortening or elevation of the entire ray or even the nonunion, either due to the fact that these techniques were applied incorrectly or due to their endogenous instability, led to poor results [2]. During the 1990s, the definition of different types of metatarsalgia according to the rocker concept during the gait cycle and a better understanding of the forefoot’s biomechanics helped to improve the enforcement of indications for the use of different procedures depending on the type of metatarsalgia [3-6].

Currently, the Weil osteotomy and triple Weil osteotomy are two effective procedures in the surgical treatment of third-rocker metatarsalgia. The latter is a modification of the classical technique, which was proposed by Maceira et al. [7,8], in order to achieve a controlled and coaxial with the shaft shortening of the metatarsal with a simultaneous elevation of the head, while avoiding the occurrence of floating toe [9,10].

The goal of the present comparative retrospective study was to evaluate the functional results and determine the complications of the two types of osteotomies through a series of patients who underwent surgery due to third-rocker metatarsalgia.

## Materials and methods

Between September 2015 and October 2020, a total of 71 patients were initially included. The study was conducted at the University of Patras and was institutionally approved by the Medical School under protocol No. 2004/12848. Written informed consent was obtained from all participants in the study.

Based on the classification into systemic or extra-regional, regional, and biomechanical metatarsalgia [11], out of all the patients, 12 of them had mixed clinical features of metatarsalgia. Of the other 59 patients, 11 of them suffered from mixed biomechanical metatarsalgia due to the simultaneous presence of functional and structural disorders (usual insufficiency of the first ray with concomitant deformities of the toes). In 48 patients, the metatarsalgia was caused by a single factor. In 6 cases, a systemic disease was responsible; in 9 cases, the cause was a local (regional) disease; and in the other 33 cases, the metatarsalgia had a biomechanical etiology, either due to functional (4 patients) or structural (29 patients) disorders (Table 1).

**Table 1:** The distribution of metatarsalgia in the initial study population according to the type of metatarsalgia. From a total of 71 patients, 27 who had a clinical feature of non-biomechanical metatarsalgia were finally excluded. 44 patients suffered from biomechanical metatarsalgia, due to a functional (4 patients) or structural (29 patients) disorder or a combination of both (11 patients), were finally studied.

Mixed features of metatarsalgia (systemic disease + biomechanical disorder, or regional disease + biomechanical disorder (12 patients)	
Neurologic disorder + deformities of toes	3
Neurologic disorder + forefoot cavus	1
Rheumatoid arthritis + deformities of toes	4
Metabolic disorder + hindfoot equinus	1
Tarsal tunnel syndrome + overload of the lesser toes	1
Tarsal tunnel syndrome + first ray insufficiency	1
Trauma (periarticular soft tissue injury) + first ray insufficiency	1
Metatarsalgia caused by a systemic disease (6 patients)	
Αutoimmune disease	4
Neurological disease	1
Vascular disease	1
Metatarsalgia caused by a local disorder (9 patients)	
Morton’s neuroma	4
Trauma (2 metatarsal fractures, 1 dislocation of MTP joint)	3
Osteochondritis	1
Soft tissue’s tumor	1
Biomechanical metatarsalgia caused by a functional disorders (4 patients)	
Hammer toe	2
Overlapping toe	1
Excessive subtalar joint pronation	1
Biomechanical metatarsalgia caused by structural disorders (29 patients)	
First ray insufficiency	9
First ray overload	3
Insufficiency of the lesser rays	5
Overload of the lesser rays	12
Μixed biomechanical metatarsalgia, caused by both functional and structural disorders (11 patients)	
First ray insufficiency + deformities of the toes	8
Overload of the lesser rays + excessive subtalar joint pronation	3

After the separation of the patients according to the type of metatarsalgia, all patients were initially treated conservatively. Conservative treatment included a number of measures such as shoe modification, use of metatarsal pads and molded insoles, rest, stretching exercises, anti-inflammatory medications, and corticosteroid injections. The duration of the conservative treatment protocol was six months. Failure to improve symptoms after this period was an absolute indication for surgical treatment. Thus, the patients suffering from biomechanical metatarsalgia (a total of 44) clinically presented a large and diffuse area of hyperkeratosis that spans several heads and underwent surgery. Only these patients were finally studied. The remaining patients were excluded from the study. The presence of previous surgery was a further exclusion criterion.

Of the 44 patients, 18 underwent surgery on both feet, while 26 underwent surgery on only one foot. Therefore, a total of 62 (26 right and 33 left) feet were studied.

Of all the patients with biomechanical metatarsalgia, 40 were women and 4 were men. The average age at the time of surgery was 58 years old (a range between 28 and 72).

Preoperative planning

In all patients, a preoperative anteroposterior weight-bearing radiograph of the foot was realized and was copied onto transparent template paper. Then, the harmonic formula of the peripheral parabolic curve of the metatarsals was drawn on the paper and the optimal correction was calculated according to the geometric regression described by Maestro, where M1 = M2, M3 = M2-3 mm, M4 = M3-6 mm, and M5 = M4-12 mm [12,13]. In this way, the number of metatarsals that had to be operated upon, as well as the exact length of shortening that had to be achieved, were determined.

When, according to the preoperative planning, a shortening of less than or equal to 3 mm was required, a simple Weil osteotomy was performed. For shortening bigger than 3 mm, however, a triple Weil osteotomy was performed. Therefore, two groups of patients have formed: the Weil osteotomy group (group A) and the triple Weil osteotomy group (group B).

Surgical technique

In 25 feet (40.32% of the total), the Weil osteotomy and the triple Weil osteotomy were performed in combination with another surgical technique. Thus, in eight cases, the Chevron osteotomy was performed on the first metatarsal, while in four other cases, the Scarf osteotomy and, in another one, the Keller osteotomy were performed. Furthermore, in five cases, the Akin osteotomy was done on the proximal phalanx, in three cases, the advancement of the extensor mechanism, and in four more, arthrodesis of the proximal phalanx was performed as well.

Weil osteotomy

During the Weil osteotomy, the patient was placed in a supine position and an ankle tourniquet was usually employed. Under epidural anesthesia, a longitudinal dorsal incision was performed just over the metatarsal or in the intermetatarsal space if the osteotomy was performed on adjacent metatarsals. Alternatively, a transverse dorsal incision was used in order to have access to three or more metatarsals.

The capsule of the metatarsophalangeal joint was opened either through a longitudinal incision medial to the tendon of extensor digitorum longus or by using a "T"-shaped incision with its vertical arm oriented centrally over the head of the metatarsal. Consequently, the collateral ligaments were released in order to achieve full access to the head [1].

The Weil osteotomy was performed starting 1-2 mm below the dorsal border of the articular cartilage, intra-articularly. The direction of the saw blade was as parallel as possible to the weight-bearing surface. In order to avoid the uncontrolled advancement of the osteotomy in the proximal metatarsal diaphysis and, therefore, the loss of its completion on the plantar surface, a long saw blade was used with an inclination of 10-15 degrees in relation to the weight-bearing level [1].

With the completion of the osteotomy, the metatarsal head was allowed to slide centrally. The degree of shortening was controlled on the dorsal surface of the proximal fragment and was also checked with the C-arm. The fixation of the osteotomy was done by means of two parallel or crossed smooth Kirschner wires, or screws of 2 mm, or even Herbert-type screws. Finally, with a rongeur, the dorsal bone prominence was removed from the proximal fragment in order not to obstruct the dorsal extension of the metatarsophalangeal joint.

Triple Weil osteotomy

During the triple Weil osteotomy, the patient was placed in a supine position and a thigh tourniquet was inflated at 300 mmHg. Under epidural anesthesia, a longitudinal or transverse dorsal incision was performed, according to the number of metatarsals that were to be operated upon. After the skin incision, the subcutaneous tissue was bluntly dissected by means of sterile gauze until the metatarsal heads were exposed. The toe was then held in plantar flexion and the metatarsophalangeal joint was longitudinally opened just medial to the tendon of the extensor digitorum longus. The preparation of the neck’s dorsal surface was then done through a sharp incision of the periosteum. In order to keep the metatarsal head from having a lack of blood flow and to be able to control how the head was aligned with the axis of the proximal phalanx, careful attention was paid not to damage the collateral ligaments.

**Figure 1:**
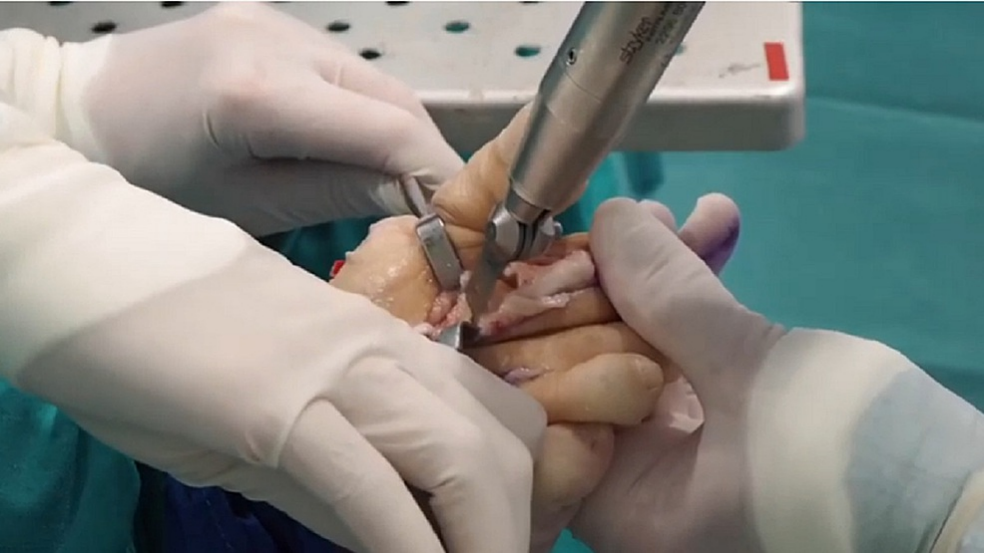
The first oblique cut of the triple Weil osteotomy, using a saw blade 15 mm long, 0.35 mm thick, and 10 mm wide, was done just proximal to the articular cartilage of the head. The orientation of the osteotomy in the sagittal plane allowed its completion plantarly, without compromising the integrity of the head.

At this point, the first cut of Maceira’s technique was obliquely performed, immediately proximal to the end of the articular cartilage. The orientation of the osteotomy in the sagittal plane was performed in such a way that after its completion it was plantarly possible. In every case, the appropriate angle of the first cut was determined by taking into account the size of the ray and the Fick angle [8]. A saw blade with a length of 15 mm, a thickness of 0.35 mm, and a width of 10 mm was used (Figure 1).

The second cut was performed in the frontal plane. The cut was performed in a vertical direction from dorsal to plantar. The shortening was calculated from the most distal edge of the proximal fragment. The third cut was exactly parallel to the first one and was performed from the dorsal edge of the proximal fragment which had arisen from the second osteotomy [14,15].

**Figure 2:**
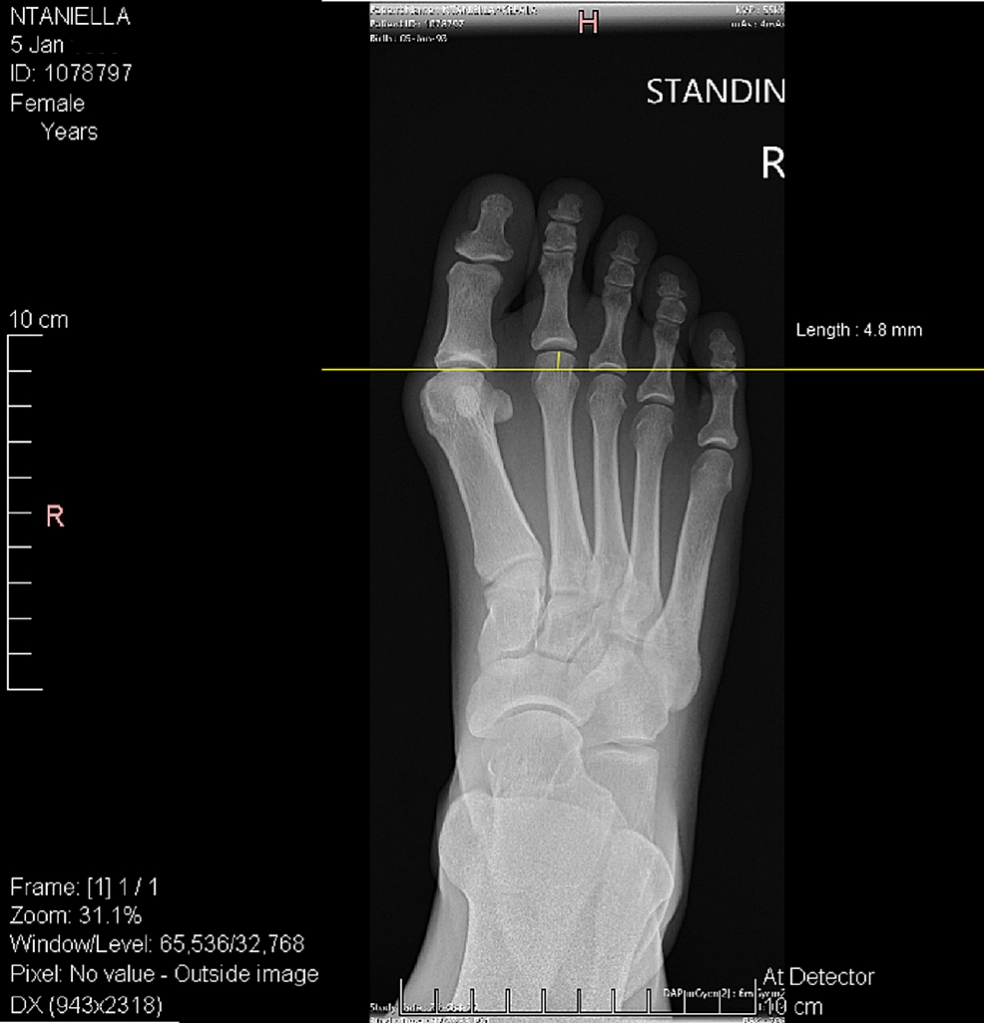
Preoperative anteroposterior weight bearing X-ray of a 28-year-old female patient which suffered by hallux valgus and third-rocker metatarsalgia due to a long second metatarsal. In this patient a Chevron osteotomy on the first metatarsal and a triple Weil osteotomy on the second metatarsal, were planned. The choice of Maceira’s technique was based on the amount of desired shortening (4.8 mm).

**Figure 3:**
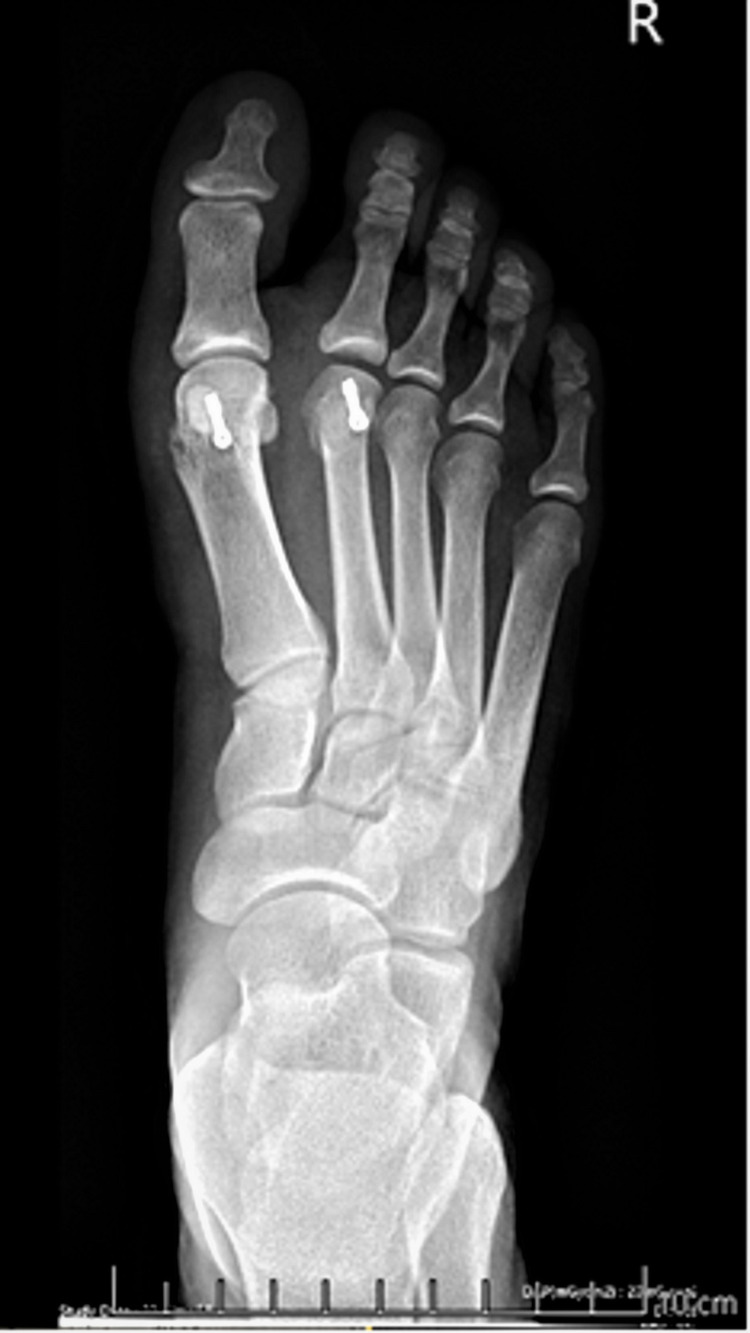
Postoperative anteroposterior weight bearing X-ray of the patient in the previous image. Two Herbert type screws were used to fix both osteotomies.

Finally, the proximal and distal fragments were repositioned and fixed either by two Kirschner wires or by Herbert-type screws (Figures 2-3). The procedure was completed with the simple closure of the skin and without prior closure of the subcutaneous tissue and the capsule. The skin was sutured without tying up the ends of the incision due to the tendency of the technique to bleed and, therefore, the necessity of proper drainage. The capsule was not sutured in order to minimize the possibility of contractures [14,15].

Clinical and radiographic evaluation

During the postoperative period, all patients were clinically and radiographically evaluated 15 days after surgery, then every month until the first four months, and then every six months. The mean follow-up was 24 months (a range between 5 and 48). The American Orthopedic Foot and Ankle Society (AOFAS) score was used for the assessment of the functional result, while the pain was assessed using the Visual Analogue Scale (VAS) [16,17].

In every follow-up, the mobility of the operated metatarsophalangeal joint was quantitatively evaluated by means of a mechanical goniometer. The measurements were taken with the patient in a supine position. The body of the goniometer was placed on the dorsal surface of the axis of motion of the examined joint, the fixed arm was placed on the middle longitudinal line of the metatarsal, and the movable arm on the middle longitudinal line of the proximal phalanx. All measurements were made, taking into account the normal range of motion of the metatarsophalangeal joint of the lesser rays. Based on the experimental studies of Jacob et al. [18], with the foot in a flat position, the transverse axis of the proximal phalanx forms an angle equal to 155° in relation to the corresponding metatarsal axis. In this position, the range of motion of the metatarsophalangeal joint is 70° of dorsal extension and 45° of plantar flexion. The "zero position" corresponded to the normal anatomical position of the metatarsophalangeal joint. The active trajectory was measured by recording the initial angle and then asking the patient to actively move the toe. The passive range of motion was measured by applying pressure to the toe at the end of its active trajectory. Based on the measurements, moderate stiffness was defined as the incapacity of the metatarsophalangeal joint to reach 75° of its range of motion, while severe dorsal stiffness was defined as the incapacity inability of the metatarsophalangeal joint to reach 25° of its range of motion.

**Figure 4:**
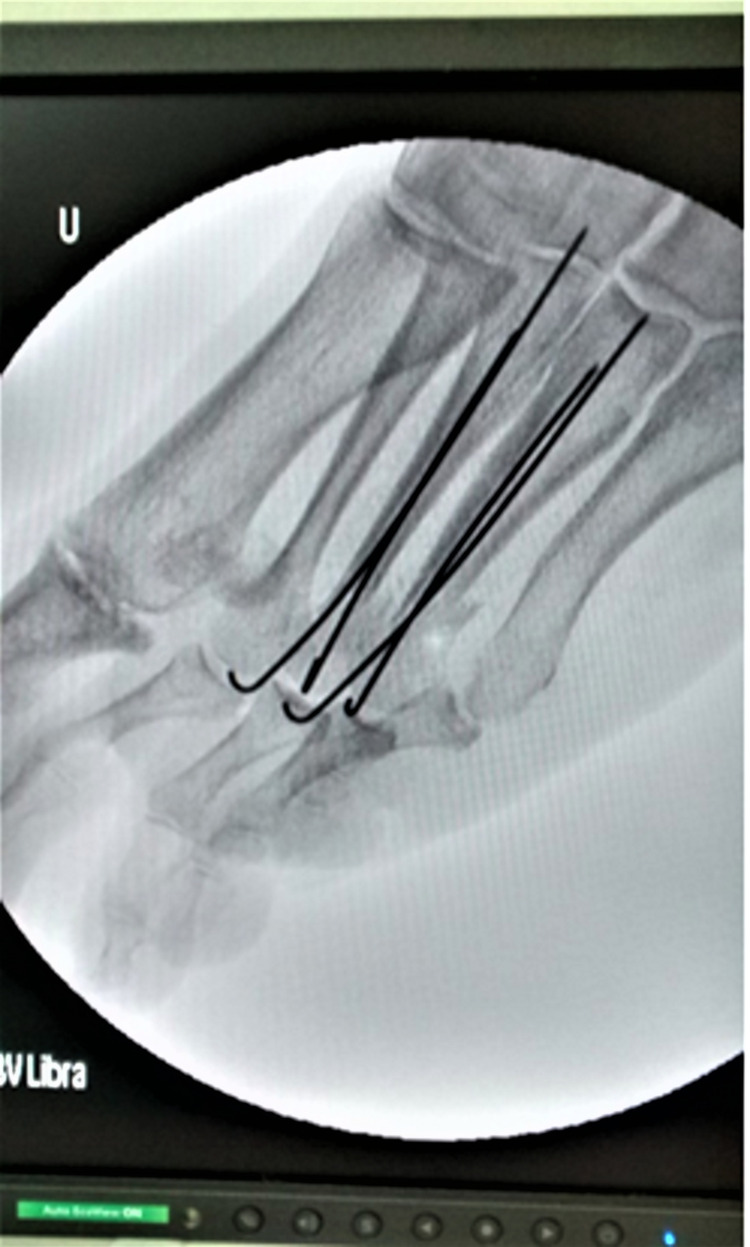
Triple Weil osteotomy in a 71-year-old female patient suffered by third rocker metatarsalgia. The fixation of the osteotomy was performed with two smooth intramedullary Kirschner wires.

The radiographic evaluation was done with a series of standardized anteroposterior and lateral X-rays. In both views, measurements of the metatarsal axis were performed in order to avoid possible deviation due to the loss of absolute stability in the cases in which the fixation was done with K-wires (Figure 4). The presence of delayed union and nonunion was also assessed.

**Figure 5:**
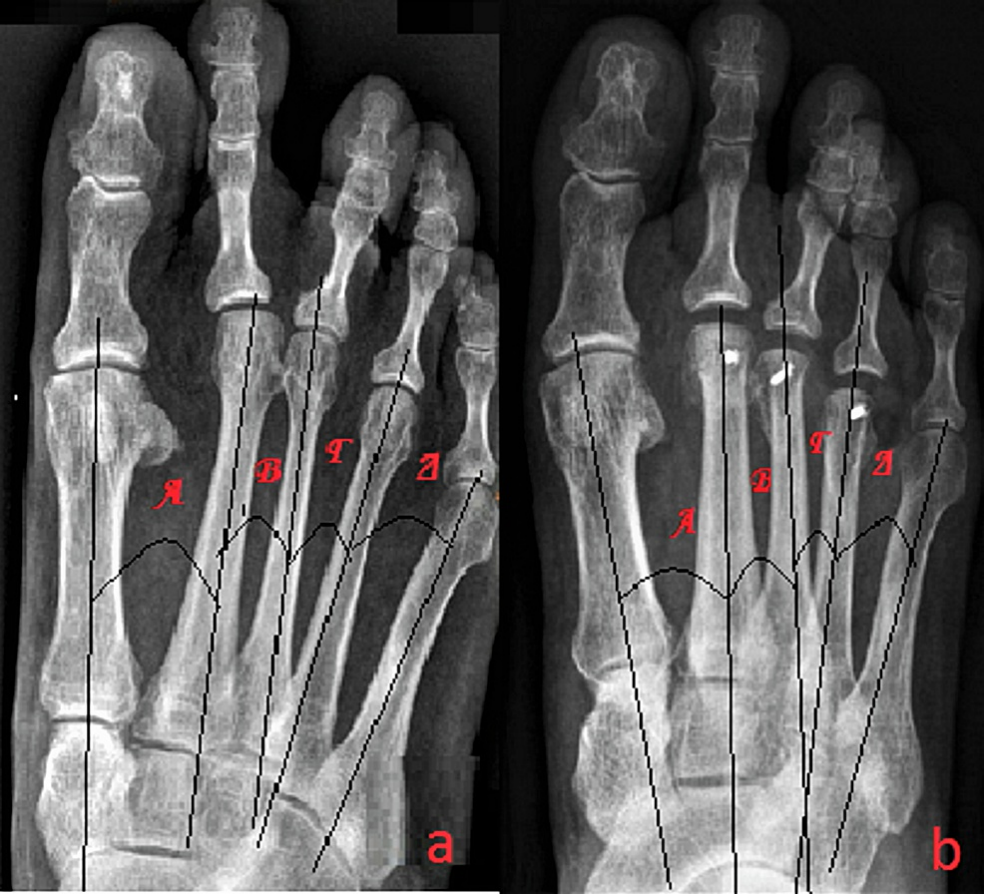
A 47-year-old patient with biomechanical metatarsalgia due to first ray insufficiency and concomitant second ray syndrome. In order to avoid possible deviation due to the loss of absolute stability at the frontal level, measurements of the angular relationship between the longitudinal axes of the metatarsals were determined. (a) Preoperative anteroposterior weight-bearing X-ray and (b) postoperative preoperative anteroposterior weight-bearing X-ray.

The anteroposterior X-rays were performed with the patient in a standing position on a platform of Plexiglas on which there was a slot for holding the X-ray cassette and by asking him to load with equal weight on both feet. Two markers made of insulating tape were placed on the backs of the patient’s heels in order to ensure the alignment of the two feet at the coronary level. The radiographic beam was perpendicularly oriented to the first tarsometatarsal joint. The center of the heel was aligned with the second ray along the vertical axis of the radiographic cassette by means of the laser guide. The source-to-film distance was equal to 1.5 m. In each patient, both preoperatively (Figure 5a) and postoperatively (Figure 5b) as well, the angular relationship between the longitudinal axes of the metatarsals was determined.

Standardized lateral X-rays were performed with the patient in a supine position and asked him to place his foot on a triangular frame made of Plexiglas, of which the vertical side had a slot for holding the X-ray cassette. In this way, the lateral X-rays were taken with the plantar surface of the foot at an angle of 30° in relation to the position of the indicating device. The orientation of the radiographic beam was always medial to lateral, and the base of the metatarsals was the focal point of the beam. The presence of deviation of the metatarsal longitudinal axis between pre- and postoperative X-rays revealed the lack of absolute stability.

Data analysis

Statistical data analysis was performed with the IBM SPSS statistical package in version 21 (Superior Performance Software System). The data were considered not normally distributed because of the small number of feet in each group. Medians and interquartile ranges were accordingly determined as descriptives. U-tests were performed in order to test for differences between group A and group B (AOFAS, VAS, and radiographic measurements). U-tests were also applied for or categorized into regular datasets (categorized range of motion of the second metatarsophalangeal joint, patient satisfaction). Before the onset of the study, a sample size calculation was done on the basis of the AOFAS score. Confidence intervals are conducted using the T-test. The comparative results were expressed as means ±95% confidence level.

## Results

From a total of 62 studied feet, 46 of them presented a plantar hyperkeratosis larger than 10 mm in diameter and painful to palpation. In 29 cases, preoperatively, the presence of subluxation or dislocation of the second metatarsophalangeal joint was detected.

A total of 90 osteotomies were performed: 46 simple Weil osteotomies and 44 triple Weil osteotomies. The distribution of the osteotomies according to the number of lesser metatarsals was: three in the second metatarsal, nineteen in the second and third metatarsals, fifteen in the second and fourth metatarsals, and three in the second, third, and fourth metatarsals (Table 2).

**Table 2:** The distribution of the osteotomies in relation to the number of the lesser metatarsals.

Metatarsal	Weil osteotomy	Triple Weil osteotomy
2°	14	10
2°–3°	19	21
3°–4°	9	10
2°–3°–4°	4	3
Total: 90 osteotomies	46	44

The average time required to perform each individual osteotomy was also measured. The average time for the Weil osteotomy was equal to 22.8 minutes, while the corresponding time for the triple Weil osteotomy was equal to 31.5 minutes. The average shortening on the second metatarsal was 5.5 mm, with a maximum shortening of 12 mm. On the third metatarsal, the average shortening was 3.5 mm, with a maximum shortening of 10 mm. Respectively, on the fourth metatarsal, the average shortening was 2.5 mm, with a maximum shortening of 6 mm.

In a case in the Weil osteotomy group, the screw had to be removed due to irritation of soft tissues. In consideration of bone healing, we would not recommend the removal of the screw before three months after the surgery. In group A, preoperatively, the average AOFAS score was 51/100 (Table 3). In the same group, postoperatively, the average AOFAS score was 89/100 (Table 4). Preoperatively, in group B, the corresponding values were 50/100 (Table 3), and postoperatively, 93/100 (Table 4).

**Table 3:** Preoperative clinical data for Weil and triple Weil osteotomy. Md: average, IQR: interquartile ranges, AOFAS: American Orthopedic Foot and Ankle Society, VAS: visual analog scale.

	Md	IQR	p-value
Weil osteotomy			
Preoperative AOFAS	51	24	0.802
Preoperative VAS	7.8	5	0.851
Triple Weil osteotomy			
Preoperative AOFAS	50	22	0.793
Preoperative VAS	8.2	3.8	0.754

**Table 4:** Postoperative clinical data for Weil and triple Weil osteotomy. Md: average, IQR: interquartile ranges, AOFAS: American Orthopedic Foot and Ankle Society, VAS: visual analog scale.

	Md	IQR	p-value
Weil osteotomy			
Postoperative AOFAS	89	18	0.925
Postoperative VAS	1.3	3.1	0.889
Triple Weil osteotomy			
Postoperative AOFAS	93	20	0.892
Postoperative VAS	1.7	3.5	0.865

The overall mean of the preoperative VAS score was 8/10, while the overall mean of the postoperative VAS score was 1.5/10. In group A, the mean preoperative VAS score was 7.8/10 (Table 3), while the mean postoperative VAS score was 1.3/10 (Table 4). The corresponding values in group B were 8.2/10 (Table 3) and 1.7/10 (Table 4), respectively.

In order to avoid a simple interpretation of the AOFAS score and to achieve a complete interpretation of the values ​​obtained from each patient, regulatory data were used as benchmarks [16]. Thus, scores of between 90 and 100 points were considered an "excellent" result, while scores of 70 to 90 points were considered a "good" result. The "moderate" result corresponded to a score between 70 and 50 points, while for an overall score below 50 points, the result was considered "poor" [16].

**Figure 6:**
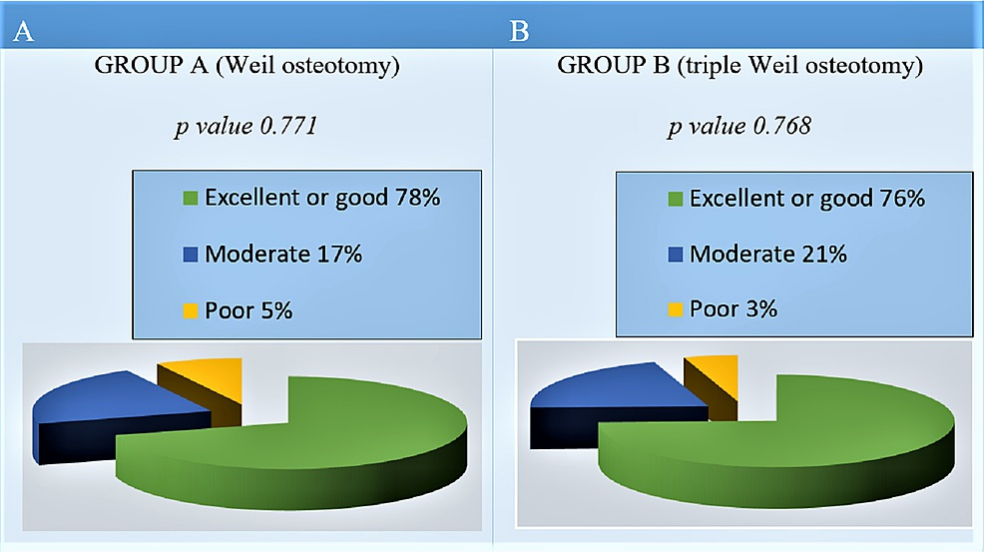
Patient satisfaction according AOFAS score, at the final follow-up. (A) Group A (Weil osteotomy) and (B) group B (triple Weil osteotomy).

Based on the above, in group A, 78% of patients had an excellent or good result, 17% had a moderate result, and 5% had an unsatisfactory one (Figure 6A). In group B, 76% of patients presented an excellent or good result, 21% presented a moderate result, and 3% an unsatisfactory one (Figure 6B).

**Figure 7:**
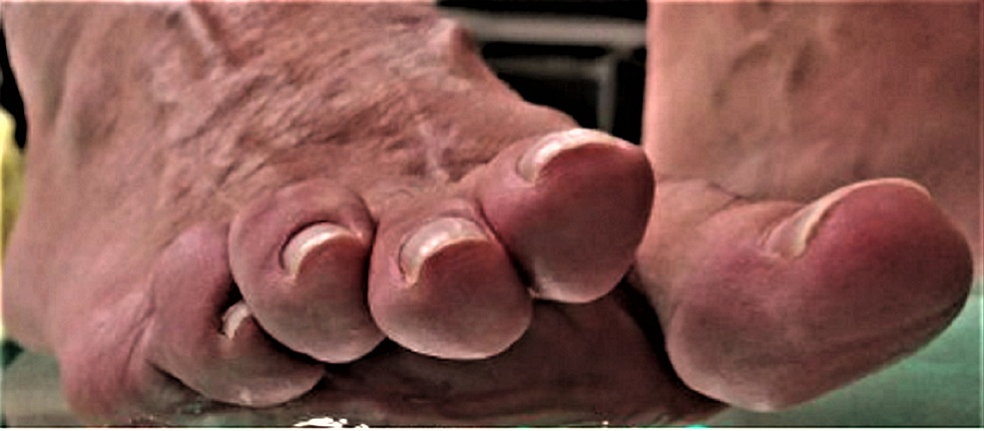
In group A (Weil osteotomy) the overall incidence of floating toe was equal to 28.3% of all osteotomies (13 osteotomies out of 46). In group B (triple Weil osteotomy), the corresponding incidence of the complication was equal to 6.8% of the total osteotomies (3 osteotomies in 44). In group B, all cases of floating toe were associated with a previous superficial infection.

Regarding the complications, in group A, the following were notified (Table 5): (a) recurrence of metatarsalgia in three patients, one of whom had severe symptoms of second-rocker metatarsalgia, (b) moderate or severe dorsal stiffness in 28 osteotomies, and (c) floating toe in 13 osteotomies (Figure 7). Ηowever, it is worth noting that, in group A, half of the patients who developed a floating toe in the last follow-up had an AOFAS score of >80.

**Table 5:** Percentage of complications in both groups.

Group A (Weil osteotomy)	
Recurrence of metatarsalgia	7.3%
Moderate or severe dorsal stiffness	60.9%
Floating toe	28.3%
Group B (triple Weil osteotomy)	
Recurrence of metatarsalgia	2.5%
Floating toe	6.8%
Delayed union	15.9%
Superficial infection	25.6%

In group B, the complications were (Table 5): (a) recurrence of metatarsalgia in one patient, (b) floating toe in three osteotomies, (c) delayed union in seven osteotomies, and (d) superficial infection in four patients, treated successfully with antibiotics per os. It is noted that, in group B, of the patients who developed floating toes, a percentage equal to 60% in the last follow-up had an AOFAS score of >80. The fact that only in group B were observed cases of superficial infection may come after a longer surgical time, which was required on average to perform the triple Weil osteotomy.

## Discussion

The Weil osteotomy is a popular and technically easy procedure in the surgical treatment of third-rocker metatarsalgia. Despite the numerous studies with good results [19,20], high complication rates are frequently associated with this osteotomy. A floating toe is the most common complication, with a reported incidence of up to 36% [21]. Indeed, the Weil osteotomy, due to the plantar translation of the metatarsal head relative to the longitudinal axis of the metatarsal and the dorsal displacement of the tendons of the interosseous relative to the center of rotation of the metatarsophalangeal joint, can lead to dorsal stiffness or even loss of contact of the toe with the ground during the third rocker of the gait [22,23]. Thus, the tendons of the interosseous muscles, which normally act as plantar flexors of the metatarsophalangeal joint, are located in a new position dorsally to the axis of rotation of these joints, resulting in attenuation of their action or their transformation into extensors for the metatarsophalangeal joint [22,23]. This fact becomes especially important when the shortening of the metatarsal is greater than 3 mm.

Based on the above remark, Monteagudo and Maceira described a modified technique that aims to recreate a more physiological metatarsal and to preserve the position of the tendons of the interosseous in relation to the longitudinal axis of the metatarsophalangeal joint [15]. The pathogenesis of floating toe, however, seems more complex. Several authors agree that the occurrence of this complication after a distal metatarsal shortening osteotomy is also due to a significant extent to the relaxation of the plantar soft tissues [23].

Our study attempted to shed light on the advantages and disadvantages of each osteotomy and to compare the two techniques in terms of their complications. In our series, we observed that the dorsal stiffness of the metatarsophalangeal joint was the commonest complication (60.9%), while a floating toe occurred in a high percentage (28.3%). In group B, the percentage of occurrences of floating toes was equal to 6.8% of all the performed triple-cut osteotomies. This seems to be associated with the greater performed amount of shortening because of the complex propulsive metatarsalgia and the concomitant subluxation of the metatarsophalangeal joint [24].

Furthermore, an important observation is that in group B, all cases of the floating toe were combined with previous superficial infections. The infection appeared to be associated with the longer and more laborious surgical times required. Finally, the presence of cases of delayed union in group B and the simultaneous absence of this complication in group A leads us to the conclusion that the Weil osteotomy has greater endogenous stability compared to the triple Weil osteotomy [25,26].

However, the study has some limitations. First, this is a retrospective case series report. Consequently, future prospective randomized studies in order to examine patients with isolated propulsive metatarsalgia and to compare the effects of the two ostomies are needed. Another limitation is that in our study there were cases in which simultaneous operations were performed (25 feet, a percentage equal to 40.32% of the total). This is because many patients with third-rocker metatarsalgia also suffer from first-ray disorders. However, concurrent surgery in both the first and lesser rays can alter the biomechanics of the foot, the results, and also the complications [10,25].

## Conclusions

The results of our study showed that both Weil osteotomy and triple Weil osteotomy are effective procedures in the surgical treatment of patients suffering from third-rocker metatarsalgia. The risk of a floating toe is higher with the Weil osteotomy and especially increases when a shortening greater than 3 mm is required in order to restore the harmonic type of the distal metatarsal parabola. Therefore, to avoid this complication, triple Weil osteotomy is preferred in these cases.

For both osteotomies, the correct preoperative planning, which must be done on weight-bearing X-rays, is of paramount importance for the outcome. Furthermore, in the appearance of the dorsal stiffness of the metatarsophalangeal joint, the healing of the surgical wound and also of the soft tissues has an important role.
